# Intrinsically Disordered Regions Define Unique Protein Interaction Networks in CHD Family Remodelers

**DOI:** 10.1096/fj.202402808RR

**Published:** 2025-05-15

**Authors:** Mehdi Sharifi Tabar, Chirag Parsania, Caroline Giardina, Yue Feng, Alex C. H. Wong, Cynthia Metierre, Rajini Nagarajah, Bijay P. Dhungel, John E. J. Rasko, Charles G. Bailey

**Affiliations:** ^1^ Faculty of Medicine & Health The University of Sydney Camperdown New South Wales Australia; ^2^ Cancer & Gene Regulation Laboratory Centenary Institute The University of Sydney Camperdown New South Wales Australia; ^3^ Centre for Rare Diseases & Gene Therapy Centenary Institute The University of Sydney Camperdown New South Wales Australia; ^4^ Cell & Molecular Therapies Royal Prince Alfred Hospital Camperdown New South Wales Australia; ^5^ School of Medical Sciences, Faculty of Medicine & Health The University of Sydney Camperdown New South Wales Australia

**Keywords:** aggregation‐prone regions, chromatin remodelers, DNA helicase, intrinsically disordered regions, protein–protein interactions, transcription

## Abstract

Chromodomain helicase DNA‐binding (CHD) enzymes play a pivotal role in genome regulation. They possess highly conserved ATPase domains flanked by poorly characterized and intrinsically disordered N‐ and C‐termini. Using mass spectrometry, we identify dozens of novel protein–protein interactions (PPIs) within the N‐ and C‐termini of human CHD family members. We also define a highly conserved aggregation‐prone region (APR) within the C‐terminus of CHD4 which is critical for its interaction with the nucleosome remodeling and deacetylase (NuRD), as well as ChAHP (CHD4, activity‐dependent neuroprotective protein (ADNP), and HP1γ) complexes. Further analysis reveals a regulatory role for the CHD4 APR in gene transcription during erythrocyte formation. Our results highlight that the N‐ and C‐termini of CHD chromatin remodelers shape protein interaction networks that drive unique transcriptional programs.

## Graphical abstract

The protein–protein interaction (PPI) networks were mapped for the intrinsically disordered N‐ and C‐termini of nine CHD family members. AlphaFold Multimer was used to predict interaction interfaces, with a focus on the interaction between the CHD4 C‐terminus and GATAD2B. We defined an aggregation‐prone region (APR) in the C‐terminus of CHD4 that mediates interactions with the GATAD2B‐NuRD (nucleosome remodeling and deacetylase) complex and ChAHP complex (via activity‐dependent neuroprotective protein (ADNP)). . Disruption of this APR‐mediated interaction using peptidomimetics can impact red blood cell differentiation (erythropoiesis) in vitro.

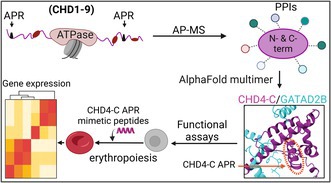

## 1 Introduction

In eukaryotes, the basic unit of chromatin is the nucleosome, which consists of 147 bp of DNA wrapped around an octamer of histone proteins [1]. The nucleosomal structure restricts the DNA accessibility of various DNA processing complexes involved in transcription, replication, and repair [2, 3]. The DNA helicase/ATPase domain of chromatin remodeler proteins hydrolyzes ATP to distort histone‐DNA interactions and thereby controls the accessibility of DNA to various DNA processing complexes [4, 5, 6, 7].

Chromatin remodelers are categorized into different families that include SWI/SNF, ISWI, INO80, and chromodomain helicase DNA‐binding (CHD) protein members [4]. The CHD family remodeling proteins are mainly involved in nucleosome assembly and organization during replication and transcription. The human CHD family contains nine members categorized into three subfamilies based on their sequence identity, domain architecture, and functional properties: subfamily I (CHD1‐2), subfamily II (CHD3‐5), which are found in the nucleosome remodeling and deacetylase (NuRD) and the ChAHP complexes [8, 9, 10, 11], and subfamily III (CHD6–9) [12, 13]. Only CHD1 is found in yeast, and the expansion of the CHD family in higher eukaryotes allows for more specialized and finely tuned regulation of chromatin dynamics and gene expression. The interaction of remodeling proteins with various chromatin binding factors expands their capacity to fine‐tune gene expression programs in many biological processes. However, owing to the large size of CHD proteins and their high affinity for nucleosomal DNA, protein interaction studies have been technically challenging. The rigorous identification of new binding partners will facilitate characterization of biological functions within and between CHD subfamilies, which may occur in a tissue‐ or context‐specific manner.

All CHD proteins belong to the superfamily 2 ATPase family, which consists of two lobes: DExx and HELICc [14, 15]. These lobes form an active‐site cleft for ATP hydrolysis that produces the required energy for DNA translocation. Additionally, all CHD members contain tandemly arranged chromatin organization modifier (chromo) domains located before the ATPase domain, which are primarily involved in binding methylated lysine residues on histones. The central chromo and helicase domains of CHDs are flanked by poorly characterized N‐ and C‐termini that contain aggregation‐prone regions (APRs) and/or intrinsically disordered regions (IDRs). These regions engage in both transient and stable protein interactions and mediate phase separation of chromatin remodelers in various biological processes [16].

Here, we utilized affinity purification followed by mass spectrometry (AP‐MS) to map the mutual and unique interaction partners of the N‐ and C‐termini of all CHD members [17]. We identified dozens of novel interactions that are either unique or common to different CHD family members. Using AlphaFold Multimer, we assessed the structural basis of several candidate interactions. By integrating our data from AP‐MS and AlphaFold Multimer, we identified and characterized a CHD4‐specific APR that can mediate interaction of CHD4 with the NuRD and ChAHP complexes in a mutually exclusive manner. We then examined the functional consequences of disrupting this APR in an in vitro red blood cell differentiation model.

## 2 Materials and Methods

### 2.1 Plasmid Constructs

All human orthologues of CHD protein fragments were constructed as GeneBlocks (Integrated DNA Technologies) and were cloned into pcDNA3.1(+) mammalian expression vectors. Each CHD fragment was tagged at the N‐terminus with the FLAG epitope. All vectors are available on request.

### 2.2 Design of APR Peptides

CHD4‐APR was conjugated to HIV‐1 Tat protein nuclear localization signal (NLS; YGRKKRRQRRR) to enhance cell permeability. A peptide representing a stretch of seven alanines was also conjugated to Tat and used as a control. Peptides were synthesized to at least 85% purity by HPLC at Mimotopes, Australia.

### 2.3 Cell Culture

G1E‐ER4 is an erythroid progenitor cell line derived from murine embryonic stem cells that stably expresses a fusion protein combining GATA‐1 fused to the estradiol receptor ligand binding domain [18]. Addition of tamoxifen (100 nM, 4‐OHT, Sigma) to the cell culture medium results in the nuclear translocation and transcriptional activation of GATA‐1, triggering erythroid differentiation over 1–2 days. G1E‐ER4 cells were cultured in Iscove's Modified Dulbecco's Medium (500 mL), supplemented with 15% (v/v) FBS, glutamine, penicillin/streptomycin, CHO Kit ligand‐conditioned medium (3 mL, generated in‐house from CHO‐KLS cells), 1‐Thioglycerol (6.2 μL, M6145, Sigma) and mouse erythropoietin (EPO, carrier‐free, 2 U/mL, #587608, BioLegend). Cells were maintained in a humidified incubator with 5% CO_2_ at 37°C. Expi293F cells were cultured to a density of 1.5 × 10^6^ cells/mL in Expi293 Expression Medium (Thermo Fisher Scientific, USA) on a horizontal orbital shaker (130 rpm).

### 2.4 Transfection and In Vitro Transcription/Translation

Polyethylenimine (PEI) was used as a transfection agent (Polysciences, Warrington, PA, USA). The DNA (4 μg) was initially diluted in 200 μL of PBS and then PEI (8 μL, 1 mg/mL) was added, followed by vortexing and then incubation for 20 min at room temperature. The mixture was added to the cells cultured in a 12‐well plate. Cells were then incubated with shaking for ~72 h at 37°C, 5% CO_2_, in a humidified incubator. For the Co‐IP experiment, the TNT Quick Coupled Transcription/Translation System was used (Promega, #L1170).

### 2.5 APR Treatment for Aggregation Analysis

Cell pellets were lysed using a buffer (500 μL) consisting of 50 mM Tris–HCl, 150 mM NaCl, 0.5% (v/v) IGEPAL, pH 7.5, 1× protease inhibitor cocktail (Sigma‐Aldrich), 1 mM DTT, and 1 μL Pierce Universal Nuclease (Thermo Fisher Scientific). Subsequently, the cells were sonicated for 5 cycles, 1 min ON/30 s OFF. Following sonication, the total lysate was collected, and the remaining lysate was portioned into new 1.5 mL Eppendorf tubes. A known concentration of APR‐containing peptides was added to each tube, and the mixture was incubated for 30 min at 4°C. The tubes were then centrifuged at 20 000 *g* for 30 min to separate the soluble and insoluble fractions.

### 2.6 3‐(4, 5‐Dimethylthiazolyl‐2)‐2,5‐Diphenyltetrazolium Bromide Assay

The 3‐(4, 5‐dimethylthiazolyl‐2)‐2,5‐diphenyltetrazolium bromide (MTT) assay is a colorimetric method used to assess cell viability and proliferation by measuring the metabolic activity of cells. The MTT assay involves NAD(P)H‐dependent cellular oxidoreductase enzyme that converts the yellow tetrazolium MTT into insoluble formazan. G1E‐ER4 cells seeded overnight in a 96‐well plate then were treated with a range of either APR peptides or CTRL peptides (10–150 μM). Two days post‐APR treatment, MTT was diluted in serum‐free RPMI‐1640 to a final concentration of 0.5 mg/mL and added to wells. Cells were then incubated for a minimum of 3 h at 37°C; quenching solution was added to a 1:1 ratio, and absorbance was read at OD = 590 nm.

### 2.7 Apoptosis Assay

G1E‐ER4 cells (2.5 × 10^5^) were treated with 25 μM of CHD4‐APR or control APR peptides for 2 h. Cells were then treated with tamoxifen (100 nM) for 28 h, followed by labeling with Annexin‐APC (BD Biosciences, #550475) and DAPI as per manufacturer's instructions (Thermo Fisher Scientific). Flow cytometry was then performed to determine the percentage of live, apoptotic, or dead cells on an LSR Fortessa (BD Biosciences).

### 2.8 Sample Preparation for Nano‐LC–MS/MS Analysis

FLAG‐tagged CHD protein domain pull‐down experiments were conducted in triplicate, along with nine replicates of FLAG‐containing empty vector pull‐downs to minimize false positives. Nuclear fractions were lysed in 800 μL of the same lysis buffer as described above. Lysates were incubated with 20 μL of FLAG antibody‐conjugated beads (Sigma‐Aldrich) for 2 h. Subsequently, a series of five washes were performed: three washes with a buffer containing 200 mM NaCl, 50 mM Tris–HCl, and 0.5% (v/v) IGEPAL at pH 7.5, and two washes with the same buffer without IGEPAL. Affinity‐purified proteins were subjected to on‐bead trypsin digestion using 20 μL of digestion buffer comprising 2 M urea freshly dissolved in 50 mM Tris–HCl, 0.5 mM DTT, 100 ng trypsin (Promega, #L1170), with the incubation carried out at 30°C–35°C for 2 h. The beads were then collected, and the supernatant was transferred to LoBind tubes. Following this, the beads were resuspended in 20 μL of 2 M urea containing 10 mM iodoacetamide in the dark for 20 min. The supernatant was transferred back to the previous tube and incubated at 30°C for 16 h. On the following day, the tryptic peptides were acidified to a final concentration of 2% (v/v) with formic acid and desalted using StageTips (Thermo Fisher Scientific). LC–MS/MS analysis was conducted using an UltiMate 3000 RSLCnano System (Thermo Fisher Scientific) coupled to a Thermo Scientific Q‐Exactive HF‐X hybrid quadrupole‐Orbitrap mass spectrometer with a standard nanoelectrospray source. The mass spectrometer was set to a data‐dependent acquisition (DDA) mode, where each full‐scan MS1 operated in a mass scan range of 300–1600 m·z^−1^ at a resolution of 60 000. The top 10 most intense precursor ions were selected for fragmentation in the Orbitrap via high‐energy collision dissociation activation in the DDA run.

### 2.9 MS Data Analysis

To confirm CHD protein expression, an intensity‐based absolute quantification (iBAQ) method was used. This algorithm normalizes the total intensity of a specific protein by the number of identified unique tryptic peptides. This normalization accounts for differences in protein length, as longer proteins are expected to yield more tryptic peptides than shorter ones. Analysis of the raw data was conducted using MaxQuant with standard settings. Carbamidomethyl cysteine and methionine oxidation were chosen as fixed and variable modifications, respectively. Trypsin was selected as the proteolytic enzyme. The output proteingroups.txt table was further processed manually to remove heat shock, plasma membrane, ribosomal, keratin, mitochondrial, and cytoplasmic proteins. Proteins represented by at least two unique peptides were set aside for analysis in the R studio package as detailed elsewhere [19]. The Perseus algorithm was employed for imputing missing values, with proteins having two missing values being excluded from the analysis.

### 2.10 Protein Analysis and Structure Prediction

ColabFold is a freely available, open‐source implementation of AlphaFold2, designed to accelerate the prediction of protein structures and complexes. ColabFold integrates the rapid homology search functionality of MMseqs2 with the advanced prediction algorithms of AlphaFold2 or RoseTTAFold and is available at https://github.com/sokrypton/ColabFold. ColabFold employs AlphaFold Multimer, an extension of AlphaFold2, to predict protein–protein interaction (PPI) complexes. Two key metrics are generated from this analysis: the Predicted Aligned Error (PAE) and the predicted Local Distance Difference Test (pLDDT) scores to report the confidence of prediction [20, 21]. In the context of protein interaction, the PAE score serves as a metric to evaluate PPI likelihood, with low PAE values indicating a high likelihood of PPIs. The pLDDT score indicates the confidence in the predicted 3D structure, with high pLDDT values reflecting high confidence in the structural prediction [20]. Predictor of natural disorder regions (PONDR) scores were used to evaluate the IDRs. The CHD protein sequences were analyzed using the TANGO algorithm to detect APRs based on beta‐sheet aggregation tendency (as a percentage) [22]. Default physicochemical parameters were selected. APRs with scores below 20% were not considered further. Pearson correlation analysis was performed using GraphPad Prism (version 10) to assess the percent identity of protein sequences.

### 2.11 RNA Extraction and RNA‐Sequencing

G1E‐ER4 cells were cultured to a density of 2.5 × 10^5^ in 12‐well plates. CHD4‐APR (YWLLAGII‐Tat) and CTRL (AAAAAAA‐Tat) were added to the culture media, each at 10 μM final concentration. After 2 h, cells were treated with 100 μM of Tamoxifen for another 28 h, and cells were collected for RNA extraction. Cell pellets were lysed in 1 mL of TRIzol Reagent (Invitrogen). After a 5‐min incubation at room temperature, 200 μL of RNase‐free chloroform (Sigma‐Aldrich) was added, followed by vigorous mixing. The samples were further incubated for 15 min at room temperature and then centrifuged at 14 000 *g* for 15 min at 4°C to separate the phases. The uppermost aqueous layer was carefully transferred to sterile 1.5 mL Eppendorf tubes, and 500 μL of isopropanol (Sigma‐Aldrich) and 1 μL of glycogen (Sigma‐Aldrich) were added and gently mixed by inversion. The RNA samples were then incubated overnight at −20°C. On the next day, the samples were thawed and centrifuged at 12 000 *g* for 20 min at 4°C. The supernatant was discarded, and the RNA pellet was washed with 1 mL of 75% (v/v) ethanol. After further centrifugation at 12 000 *g* for 5 min at 4°C, the supernatant was removed, and the RNA pellet was allowed to air‐dry. Finally, 30 μL of RNase‐free, UltraPureTM distilled water (Gibco) was added, and the RNA samples were stored at −80°C.

### 2.12 Differential Gene Expression Analysis

After determining the raw sequence data quality using FASTQC and adaptor trimming using Cutadapt reads were then aligned to the Ensembl mouse genome (GRCm38, release 86) using the STAR aligner (version 2.7.10a) [23, 24]. Gene expression levels were quantified by generating a count matrix using the FeatureCounts command from the Rsubread package [25], and differential expression analysis was performed using the DESeq2 package in R [26]. Heatmaps were generated using R‐shiny app FungiExpresZ [27]. For gene ontology (GO) analysis, ShinyGO (version 0.8) was employed on the subset of differentially expressed genes that demonstrated significant changes (FDR < 0.05) and a log2 fold‐change exceeding 1.5 [28]. The entire list of expressed genes served as the background for this analysis.

## 3 Results

### 3.1 CHD N‐ and C‐Termini Are Highly Disordered

**FIGURE 1:**
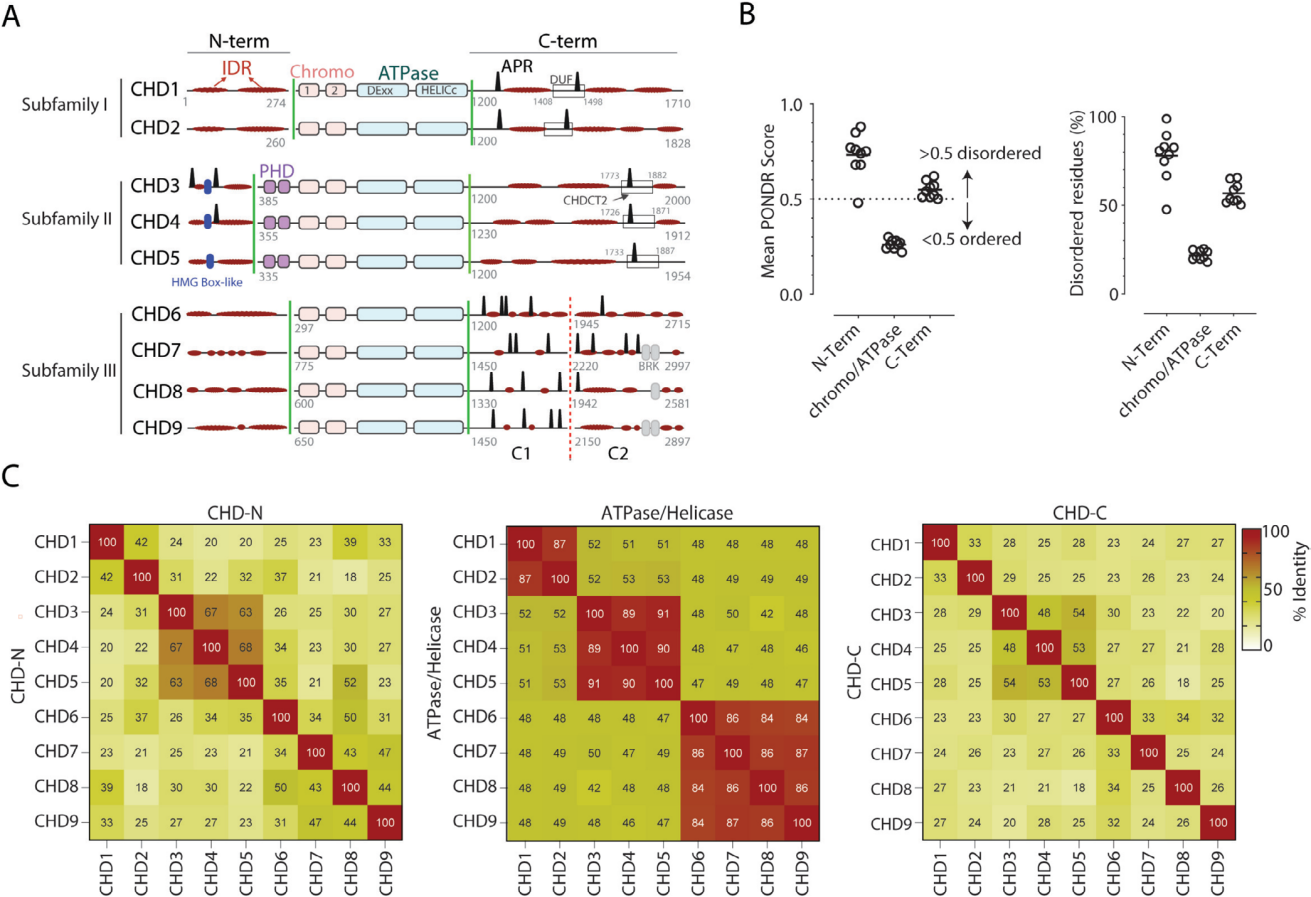
Human CHD family members exhibit both unique and shared structural characteristics. (A) Representation of the domain organization of CHD proteins, highlighting the distribution of aggregation‐prone regions (APRs, black peaks) and intrinsically disordered regions (IDRs, flat red ovals) within the N‐ and C‐termini. The domain of unknown function (DUF) and CHD C‐terminal 2 (CHDCT2) domains, which contain APR regions within the C‐termini, are represented by rectangles. Green lines denote boundaries for N‐ and C‐terminal segments used in AP‐MS analysis. The C1 and C2 segments, separated by a dotted red line, denote further truncated CHD6‐9 C‐termini. Numbers in gray refer to amino acid positions. Blue and gray ovals represent high mobility group (HMG) Box‐like and Brahma and Kismet (BRK) domains, respectively. (B) The graphs illustrate the mean PONDR score and the proportion of disordered residues (%) of the N‐terminal, ATPase, and C‐terminal domains of all CHD proteins. (C) Heatmap matrices represent Pearson correlation analysis within and between CHD subfamily domains.

Both the chromo and ATPase domains of CHD proteins are centrally located and flanked by mostly unstructured N‐ and C‐termini containing unique functional domains (Figure 1A). Only subfamily II (CHD3‐5) N‐termini contain a high mobility group Box‐like domain and tandem plant homeodomains that bind to poly(ADP‐ribose) and H3K9me3 posttranslational marks, respectively [29]; whereas the C‐termini contain a poorly defined CHD C‐terminal (CHDCT2) domain (Figure 1A). Other C‐terminal domains include a structured domain of unknown function (DUF) in CHD1 and Brahma and Kismet domains in members of CHD subfamily III (Figure 1A).

We examined the presence of IDRs within the N‐ and C‐termini, which revealed long stretches of highly disordered residues (Figure 1A). We used PONDR, a web‐based computational tool, to predict the probability of disordered regions in all CHDs. PONDR scores confirmed that both the N‐ and C‐termini are highly disordered (score > 0.5), while the chromo/ATPase domains are highly ordered (score < 0.5) (Figure 1B). Additionally, about 80% of the N‐terminal residues and 53% of the C‐terminal residues are disordered, whereas only 21% of the chromo/ATPase domain residues are disordered (Figure 1B). We also examined the number and distribution patterns of APRs in CHD proteins using TANGO, a web‐based computer algorithm for the prediction of aggregating regions in unfolded polypeptide chains (Figure 1A). While the N‐termini of subfamilies I and III are devoid of APRs, only CHD3 and CHD4 members of subfamily II contain two and one APRs within the N‐termini, respectively (Table S1). C‐terminal analysis showed that subfamilies I and III contain two or more APRs, whereas only subfamily II members possess a unique APR within their CHDCT2 domain (Figure 1A). In the central ATPase domain‐containing region of CHDs, our sequence alignment analysis reveals that there is ~87% sequence identity across various CHD proteins within each subfamily (Figure 1C, Table S1). In contrast, sequence alignment and identity analysis of the N‐ and C‐termini of CHD family proteins indicate that they are much less conserved, with sequence identity ranging from 24% to 54% (Figure 1C).

To map the CHD termini‐specific protein interaction networks, we cloned the N‐ and C‐termini of all CHDs into a mammalian expression plasmid also containing a FLAG epitope (Figure 1A). The longer C‐termini of CHD6‐9 were divided into two distinct segments, identified as C1 and C2, to facilitate expression (Figure 1A). All fragments were transiently overexpressed in Expi293 cells, the nuclear fraction was collected, and the FLAG‐tagged CHD fragments were immunoprecipitated using FLAG antibody‐conjugated beads. A FLAG‐containing empty vector was used as the control. To ensure that all fragments were expressed and purified, we evaluated the abundance of FLAG‐CHD by AP‐MS. Of 22 CHD fragments tested, 19 were detected and quantified (Figure S1). Fragments that did not show any detectable expression (i.e., CHD7‐N, CHD6‐C1 and 2) or failed to immunoprecipitate any proteins (CHD8‐C1) were excluded from further analysis.

### 3.2 The Protein Interactions of CHD Family N‐Termini

**FIGURE 2:**
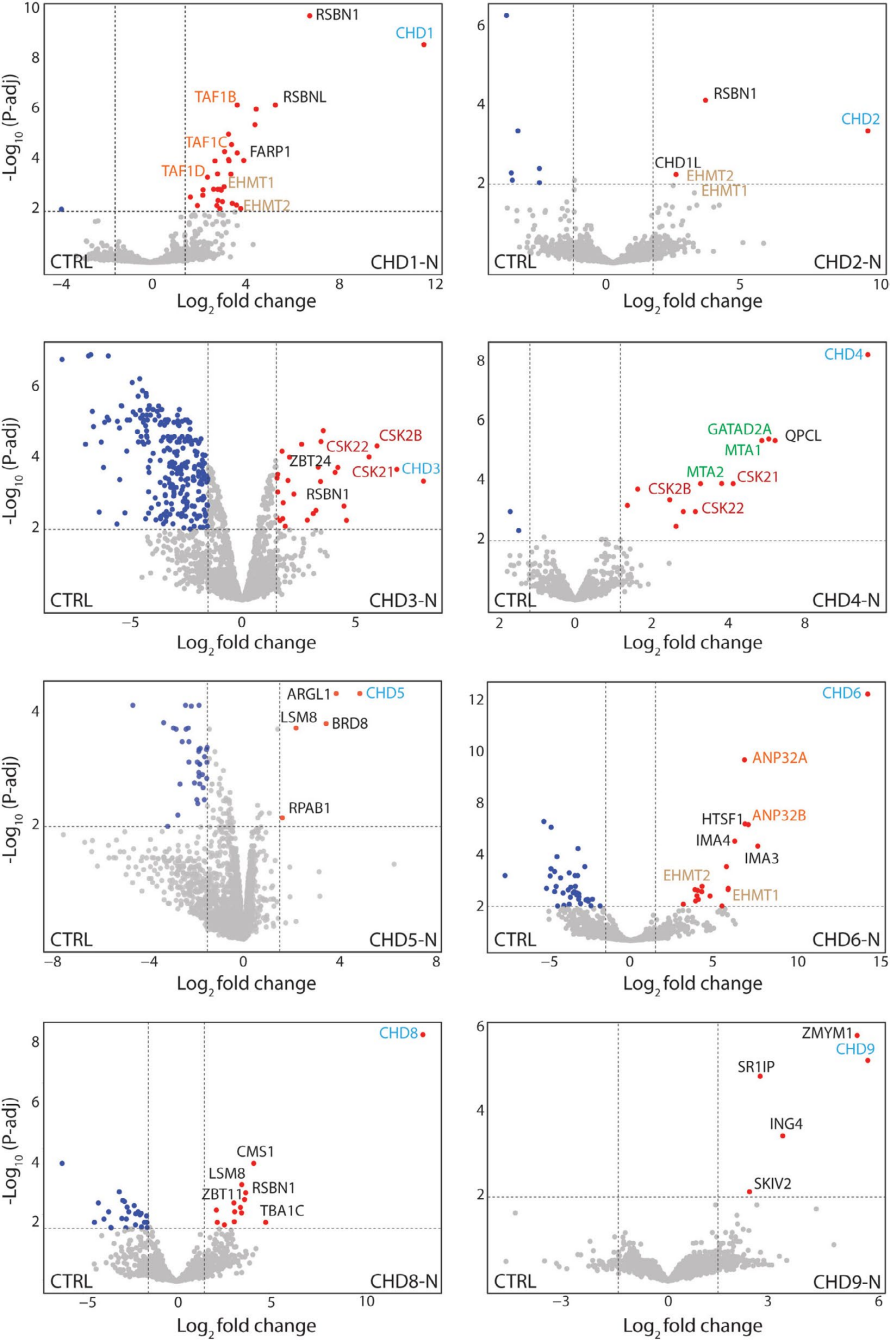
The protein interaction landscape of CHD N‐termini. Volcano plots representing affinity purification of CHDs N‐termini using FLAG antibody‐conjugated beads followed by label‐free mass spectrometry analysis (*n* = 3). CHD bait proteins are labeled in cyan, while significantly co‐enriched proteins are represented by red dots. Co‐enriched proteins associated with gene ontology terms related to chromatin and gene transcription were manually selected and labeled on the graphs. Proteins that are part of multi‐subunit complexes are represented with the same color; NuRD subunits are represented in green in the CHD4‐N panel. Significance was determined as log_2_ (fold change) > 1.5, −log_10_ (*p*‐adj) > 2; gray dots indicate proteins which were not significantly enriched.

AP‐MS analysis revealed a distinct protein interaction signature within and between some CHD proteins and subfamilies. Among the enriched proteins, we noticed a marked enrichment for several novel and known chromatin binding or modifying proteins or multi‐subunit protein complexes (Figure 2, Table S2). We manually selected candidates with GO terms related to chromatin biology and represented them on the graphs (Figure 2). For instance, the SL1 protein complex members (the general TAT‐binding associated factors [TAF1B, TAF1C, TAF1D]) were enriched with CHD1. TAFs nucleate RNAPII formation at promoter regions, and CHD1 regulates RNAPII initiation and transcription [30, 31]. The interaction might provide functional synergy to control productive transcription. The euchromatic histone lysine methyltransferase (EHMT1/EHMT2) complex, responsible for adding methyl groups to H3K9 histone residues [32, 33], was enriched with the N‐termini of various CHDs, including CHD1‐2 and CHD6. A marked enrichment of round spermatid basic protein (RSBN1) with CHD1‐3 and CHD8 was observed. RSBN1, also known as KDM9, acts as a demethylase on the histone mark H4K20me2, a DNA replication mark [33]. This provides supportive evidence for the involvement of the remodelers in coordinated gene transcription and DNA replication. We also observed the enrichment of the casein kinase II complex subunits (CSK2B, CSK21, and CSK22) [34] with the N‐termini of CHD3‐4. Other notable enrichments included bromodomain‐containing protein (BRD8) with CHD5, acid nuclear phosphoprotein (ANP32A/B) with CHD6, and zinc finger MYM‐type‐containing (ZMYM2) with CHD9, each of which is known to play roles in chromatin organization and gene expression.

### 3.3 The Protein Interactions of CHD Family C‐Termini

**FIGURE 3:**
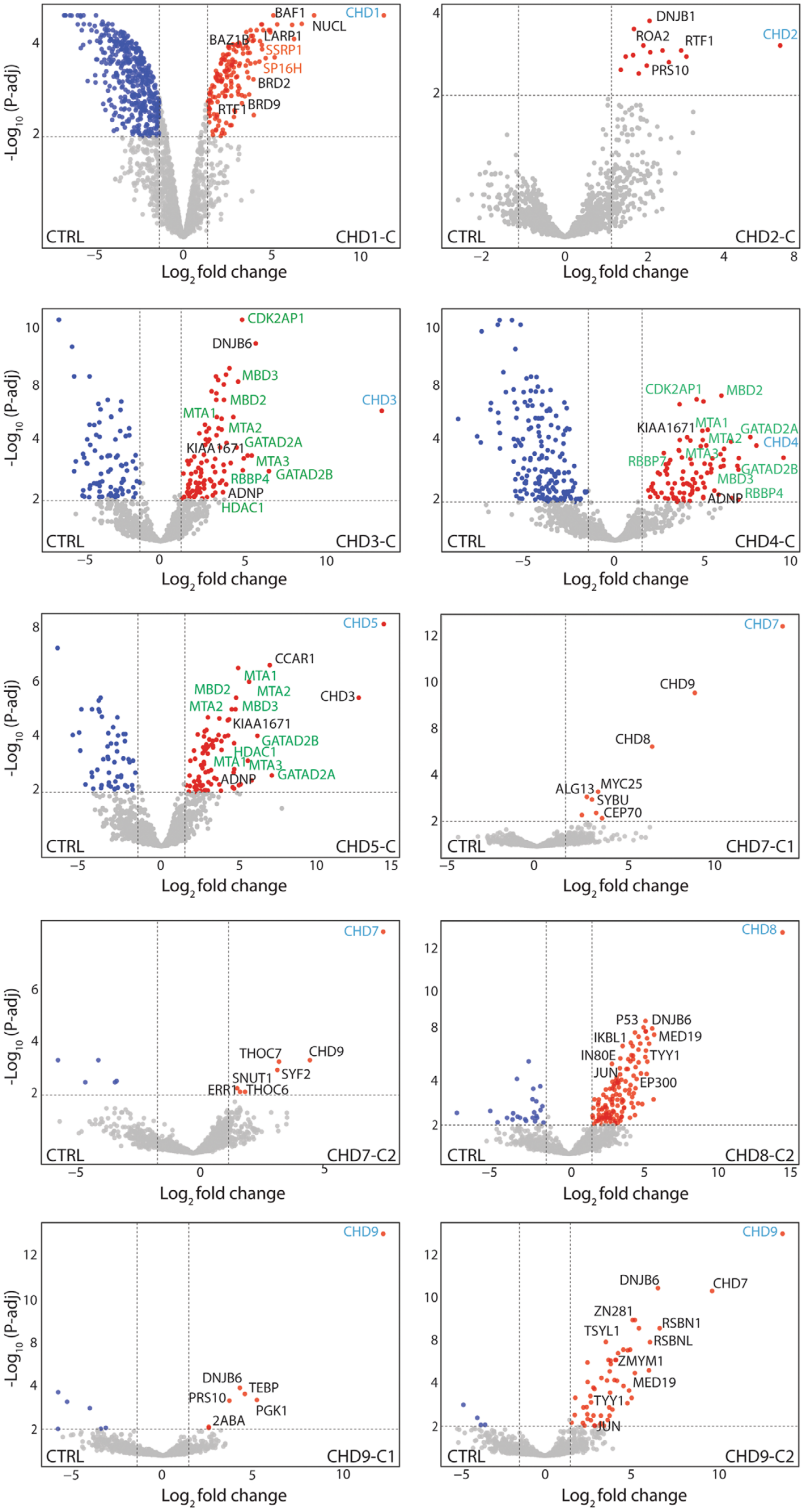
The protein interaction landscape of CHD C‐termini. Volcano plots representing affinity purification of CHDs C‐termini using FLAG antibody‐conjugated beads followed by label‐free mass spectrometry analysis (*n* = 3). CHD bait proteins are labeled in cyan, while significantly co‐enriched proteins are represented by red dots. Co‐enriched proteins associated with gene ontology terms related to chromatin and gene transcription were manually selected and labeled on the graphs. Proteins that are part of multi‐subunit complexes are represented with the same color; NuRD subunits are represented in green in the CHD3‐5C panels. Significance was determined as log_2_ (fold change) > 1.5, −log_10_ (*p*‐adj) > 2; gray dots indicate proteins which were not significantly enriched.

Analysis of CHD protein C‐terminal fragments identified additional unique and shared proteins involved in gene regulation and chromatin remodeling (Figure 3, Table S2). For example, the enrichment of barrier to autointegration factor, a core component of the SWI/SNF remodeling complex, with CHD1 is intriguing as both have been shown to localize to the promoter and enhancers of target genes [35, 36]. In addition, histone chaperones including structure‐specific recognition protein and SPT16 homolog, subunits of the FACT (facilitates chromatin transcription) complex, were enriched with the CHD1 C‐terminus. Together, CHD1 and FACT facilitate RNAPII passage through nucleosomes to aid transcription [37]. Our observations reveal the co‐enrichment of RTF1, an RNAPII‐associated protein, further validating previous findings and underscoring its pivotal role in transcriptional regulation [38] (Figure 3). Consistent with our previous findings, we observed a marked enrichment of the NuRD complex subunits and activity‐dependent neuroprotective protein (ADNP), the core subunit of the ChAHP complex, with the C‐termini of CHD3‐5 [10, 11, 39]. A notable enrichment of KIAA1671 exclusively with the C‐termini of CHD3‐5, but no other CHDs (Figure 3), may suggest a unique role for this poorly characterized protein in gene regulation and chromatin organization. ZMYM1, a lowly expressed nuclear protein, was specifically enriched with the C‐terminus of CHD9. DNAJ homolog subfamily B member, DNAJB6, was co‐enriched with the C‐termini of most CHD proteins, which suggests a possible transcriptional regulatory role for this chaperone as a stimulatory effector on ATPase activity. In summary, our analysis provides an important resource comprising both unique and shared proteins that bind via the N‐ and C‐termini of CHD family proteins.

### 3.4 Evaluation of CHD Protein Interactions With AlphaFold

**FIGURE 4:**
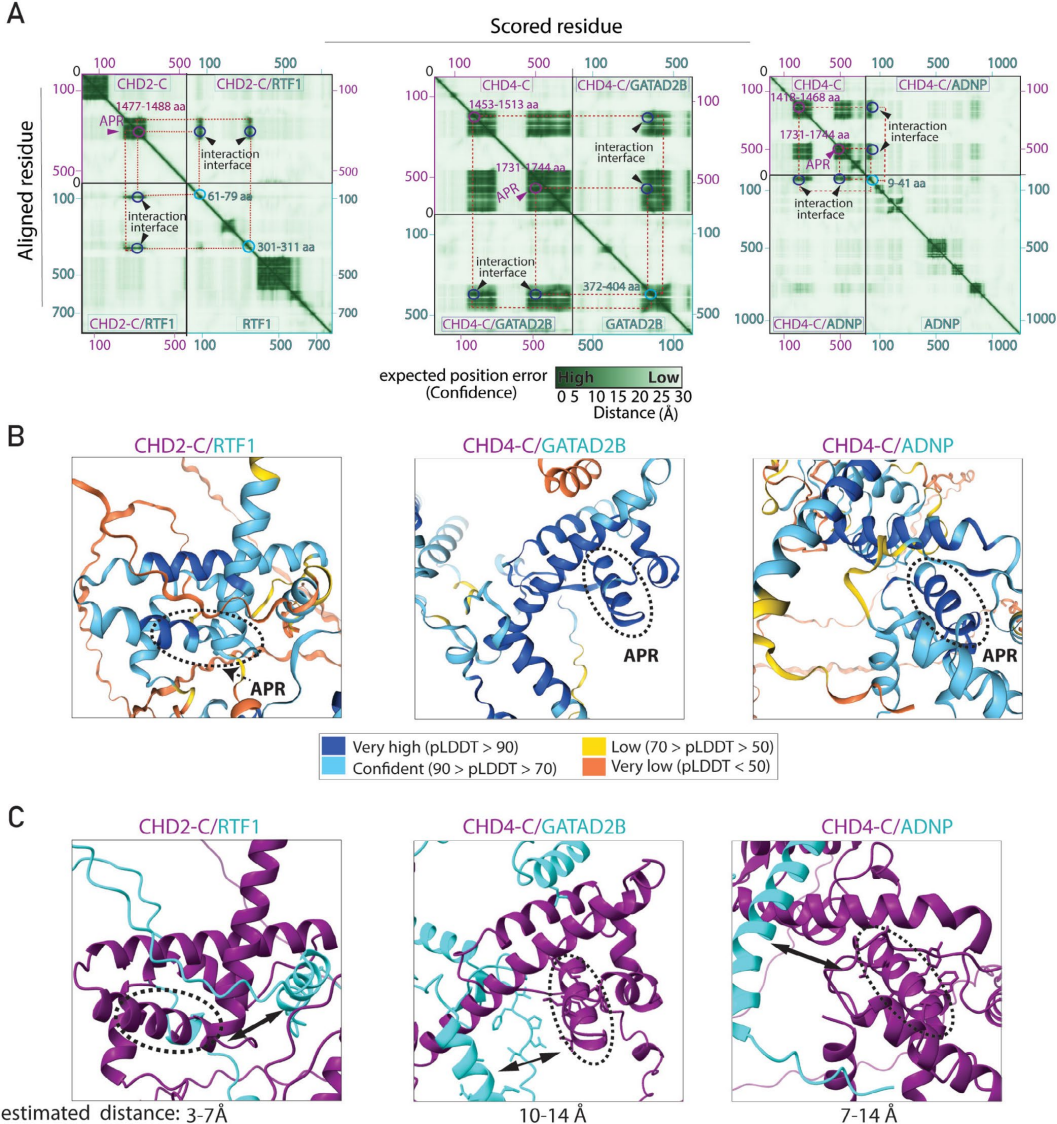
AlphaFold validation of CHD protein interactions discovered by AP‐MS. (A) Heatmaps represent the Predicted Aligned Error (PAE) score between all pairs of residues. A dark green color indicates a low PAE, signifying high reliability of the relative position of the residues. Conversely, lighter shades denote lower confidence in the positioning. Potential interaction interfaces are indicated by arrowheads. Potential interacting residues within each polypeptide are indicated in the same color as the protein label. (B) Predicted 3D structure of the identified complexes. The predicted local distance difference tool (pLDDT) score represents the confidence of the predicted structure. APRs form alpha helical structures with high pLDDT scores (highlighted with dotted circles in B and C). (C) Estimated contact distances between APRs (purple) and interacting proteins (cyan): Left, between CHD2‐C and RTF1; middle, between CHD4‐C and GATAD2B; right, between CHD4‐C and ADNP. Double‐headed arrows in (C) indicate the estimated distance between the two polypeptides.

To independently evaluate the interaction likelihood and structural basis of identified protein interactions, we utilized Alphafold Multimer, an extension of AlphaFold2 [20]. In AP‐MS, significant protein enrichment indicates a high probability of physical interaction. Therefore, we first selected several candidates that play a role in chromatin biology and exhibited strong co‐enrichment patterns with CHD proteins. These include the CHD1‐N:EHMT1, CHD5‐N:BRD8, CHD6‐N:ANP32A, CHD9‐C2:DNAJB6, CHD2‐C:RTF1, CHD4‐C:ADNP, and CHD4‐C:GATAD2B (GATA zinc finger domain‐containing 2B) complexes. AlphaFold Multimer analysis yielded very poor pLDDT scores (see Section 2) for most of these complexes, meaning that it failed to confidently predict interaction (Figure S2A,B). This is consistent with previous findings that AlphaFold cannot confidently predict 3D structure for disordered proteins and regions [40, 41]. However, AlphaFold predicted high confidence interactions (low PAE and high pLDDT scores) for several complexes such as CHD2‐C:RTF1, CHD4‐C:GATAD2B, and CHD4‐C:ADNP complexes (Figure 4A,B). When we further focused on the predicted regions as potential interacting domains, we noted that this is mainly because CHD2‐C and CHD4‐C contain relatively well‐structured but functionally poorly understood DUF and CHDCT2 domains, respectively (see Figure 1A). We then further evaluated these three complexes and observed the presence of several α‐helical structures, including APRs (indicated by dark blue circles) at the interaction interfaces (Figure 4B). AlphaFold predicted two potential interaction interfaces for the CHD2‐C‐RTF1 complex. In this complex, the CHD2‐C APR region (residues 1477–1488) can potentially interact with residues 61–79 and/or 301–311 on the RTF1 protein. For the CHD4‐C:GATAD2B complex, AlphaFold predicted two potential regions on CHD4‐C: the first region includes residues 1453–1513, and the second region includes residues 1731–1744, which encompass the APR region. These regions might interact with either residues 372–404 on the GATAD2B protein or residues 9–41 on the ADNP protein.

To investigate whether APRs are involved in the direct interaction with RTF1, GATAD2B, and ADNP, we measured the distance between APRs and the closest residues of the interacting partner using Chimera X. Our analysis suggests that the CHD2‐C interaction with RTF1 could be direct, with an estimated distance of 3–7 Å. Measurement of CHD4‐C‐specific APRs distance with both ADNP and GATAD2B suggests a distance > 7 Å. This highlights that CHD4‐C APR may not be directly involved in the interaction; however, it does not exclude the contribution of the APR to protein interaction (Figure 4C). Consistent with our previous research that established the role of APRs in PPIs [10, 39], these data reveal that the interaction of CHD2 with RTF1 and CHD4 with either GATAD2B or ADNP is likely to be mediated by the APRs located in their C‐termini. To provide biological insight, the remainder of the study focuses exclusively on the CHD4‐C APR protein interactions. Experimental validation of the CHD2‐C APR interaction with RTF1 still needs to be performed and will be the subject of future investigations.

### 3.5 APR Contributes to CHD4 Interaction With NuRD and ChAHP Complexes

**FIGURE 5:**
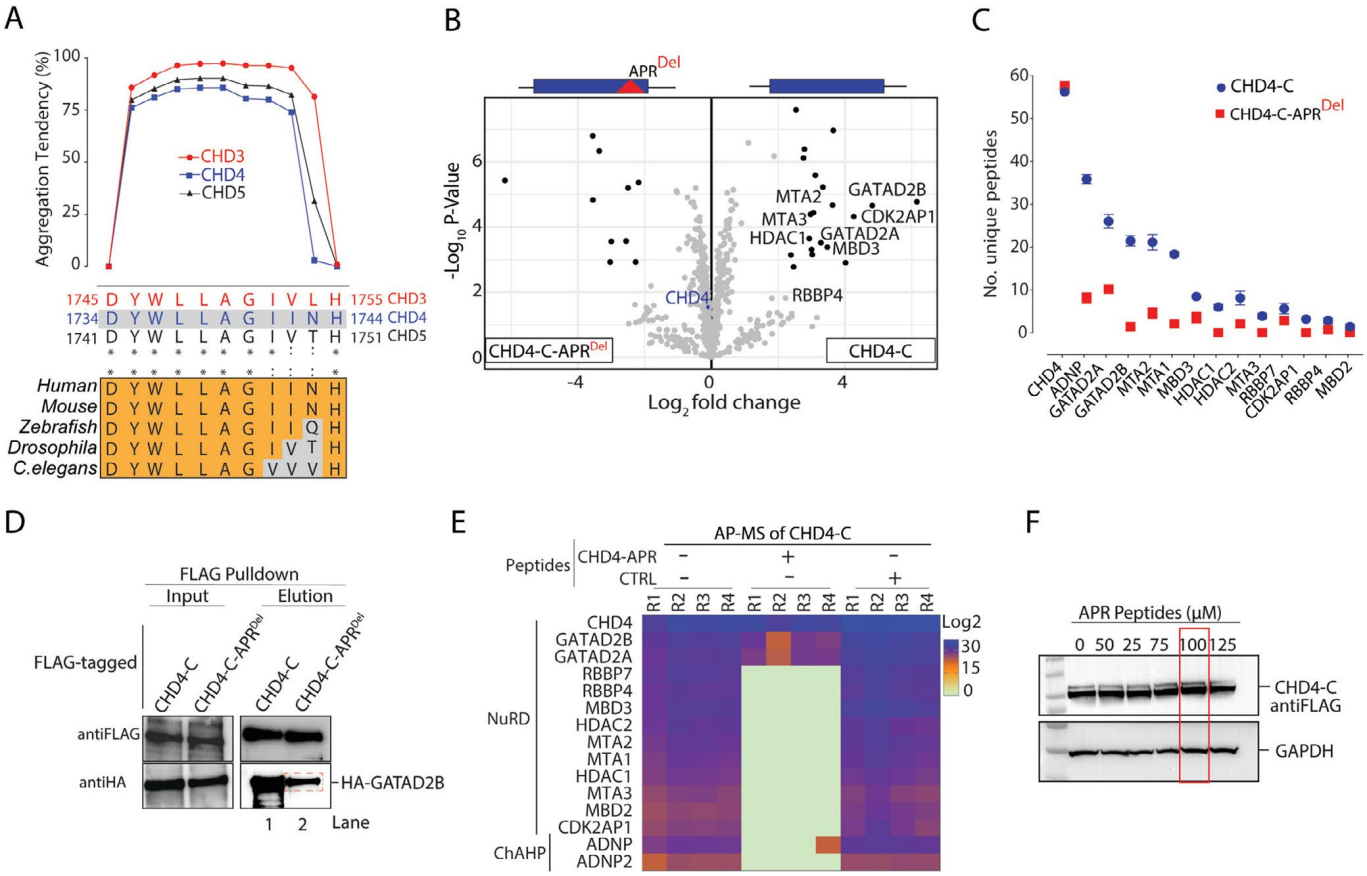
The C‐terminus of CHD4 protein harbors an APR that directly interacts with the NuRD and ChAHP complexes. (A) TANGO analysis shows β‐sheet aggregation tendency for the conserved APR within CHD3, CHD4, and CHD5. Corresponding APR residues are shown with homology within CHD3‐5 subfamily and between CHD4 orthologues. (B, C) AP‐MS for interactors of CHD4‐C and CHD4‐C‐APR^Del^ showing: (B) volcano plots of enriched proteins; and (C) the number of unique peptides of subunits of the NuRD and ChAHP complexes detected. (D) Western blots of input and elution samples from FLAG‐CHD4‐C co‐expressed with HA‐GATAD2B protein using an in vitro transcription/translation system; samples were probed with anti‐HA and anti‐FLAG antibodies. (E) Cellular lysate expressing FLAG‐CHD4‐C incubated in the presence of CHD4‐APR or CTRL peptides (100 μM) before subsequent AP‐MS. The heatmap represents intensity of detected peptides for CHD4‐C‐NuRD and ‐ChAHP complex subunits. (F) Western blots confirming consistent detection of FLAG‐CHD4‐C protein after addition of APR peptidomimetics (0–125 μM) to the cell lysates. Red box indicates the concentration used in (E).

CHD4, a core component of both NuRD and ChAHP complexes, binds GATAD2A/B in NuRD and ADNP in ChAHP. This suggests that CHD4 may contain an interface that supports its interaction with either the NuRD or ChAHP complexes. Thus, we hypothesized that the aliphatic stretch located in CHD4 (1735–1742 aa, YWLLAGII), the sole APR predicted in our TANGO analysis in the CHD4 C‐terminus, could contribute to CHD4 interaction with both ADNP and GATAD2B (Figure 5A). This motif is evolutionarily conserved in CHD4 orthologues and between CHD subfamily II members (Figure 5A). To examine the role of this putative APR in mediating PPIs, we conducted an AP‐MS experiment using FLAG‐tagged CHD4‐C and a deleted APR mutant (CHD4‐C‐APR^Del^) as bait. Our data demonstrated a pronounced enrichment of canonical NuRD subunits and ADNP with CHD4‐C, compared to the CHD4‐C‐APR^Del^ mutant (Figure 5B). CHD4 expression levels were similar in both conditions, as highlighted in the center of the volcano plot. This is also evident in the equivalent amount of unique CHD4‐C peptides identified in both experiments, whereas there is a noticeable decrease in the number of identified peptides for all NuRD subunits and ADNP (Figure 5C). To further corroborate these observations, we performed pairwise co‐immunoprecipitation of hemagglutinin (HA) epitope‐tagged GATAD2B‐C versus either FLAG‐CHD4‐C or ‐CHD4‐C‐APR^Del^, as GATAD2B is a direct binding partner of CHD4 within the NuRD complex [10, 39]. We co‐expressed FLAG‐tagged CHD4‐C or CHD4‐C‐APR^Del^ with HA‐tagged GATAD2B using an in vitro transcription and translation system. The lysates were mixed with FLAG‐beads to capture the complexes. This was followed by washing and elution with 3X‐FLAG peptides. Western blot analysis of the eluate using anti‐HA antibody revealed a reduction in GATAD2B binding to CHD4‐C‐APR^Del^ compared to CHD4‐C (lane 2 vs. lane 1). Complete abrogation of GATAD2B from CHD4 might be achieved by mutating residues within the other potential interaction interface (1453–1513 aa) predicted by AlphaFold Multimer (see Figure 4A). Together, our data provide evidence that the APR region is important for the physical interaction of CHD4 with GATAD2A/B (Figure 5C).

To rule out the possibility that the disruption of CHD4‐C protein interactions upon deletion of the APR was caused by protein destabilization, we employed an alternative strategy using PPI‐disrupting peptidomimetics. These short, synthesized peptides mimic specific protein regions and can either disrupt or competitively inhibit binding partners without affecting protein stability. Peptides containing the aliphatic stretch (referred to as CHD4‐APR) or a stretch of alanine residues (referred to as control [CTRL]), both conjugated to the HIV‐1 Tat NLS were used. In a competition assay, we overexpressed FLAG‐tagged CHD4‐C in HEK293 cells and mixed the cell lysate with either CHD4‐APR or CTRL peptides, followed by AP‐MS experiments. Comparison of co‐purified proteins from FLAG‐CHD4‐C samples treated with CHD4‐APR peptidomimetics versus CTRL peptides showed a significant depletion of most NuRD subunits and ADNP1/2 proteins in the CHD4‐APR‐treated samples, but not in those treated with CTRL peptides (Figure 5E). Consistent with the co‐IP results, the enrichment of GATAD2B was diminished but not completely disrupted. Additionally, incubation of FLAG‐CHD4‐C‐expressing cell lysates with a range of CHD4‐APR peptide doses did not reduce FLAG‐CHD4‐C protein expression (Figure 5F). This observation indicates that the APR does not affect CHD4 protein levels, and therefore, the diminished GATAD2B enrichment directly results from a disrupted PPI. This observation supports the hypothesis that the CHD4 C‐terminal APR contributes to the integration of CHD4 into the NuRD and ChAHP complexes.

### 3.6 The APR Region is Critical for the Function of the NuRD and ChAHP Complexes

CHD4 plays a critical role in regulating gene expression during erythropoiesis, the process of red blood cell formation. To study this, we utilized mouse G1E‐ER4 cells, a well‐established model of erythroid differentiation. These cells are engineered to ectopically express a fusion protein consisting of the estrogen receptor (ER) and the master transcriptional regulator of erythropoiesis, GATA1. Upon stimulation with tamoxifen, the GATA1‐ER fusion protein relocates to the nucleus, triggering the cells to undergo synchronized differentiation into erythrocytes [32]. We first examined the impact of CHD4‐APR peptidomimetics on undifferentiated G1E‐ER4 cell viability using MTT, and quantifying apoptotic cells by labeling with Annexin‐APC.

**FIGURE 6:**
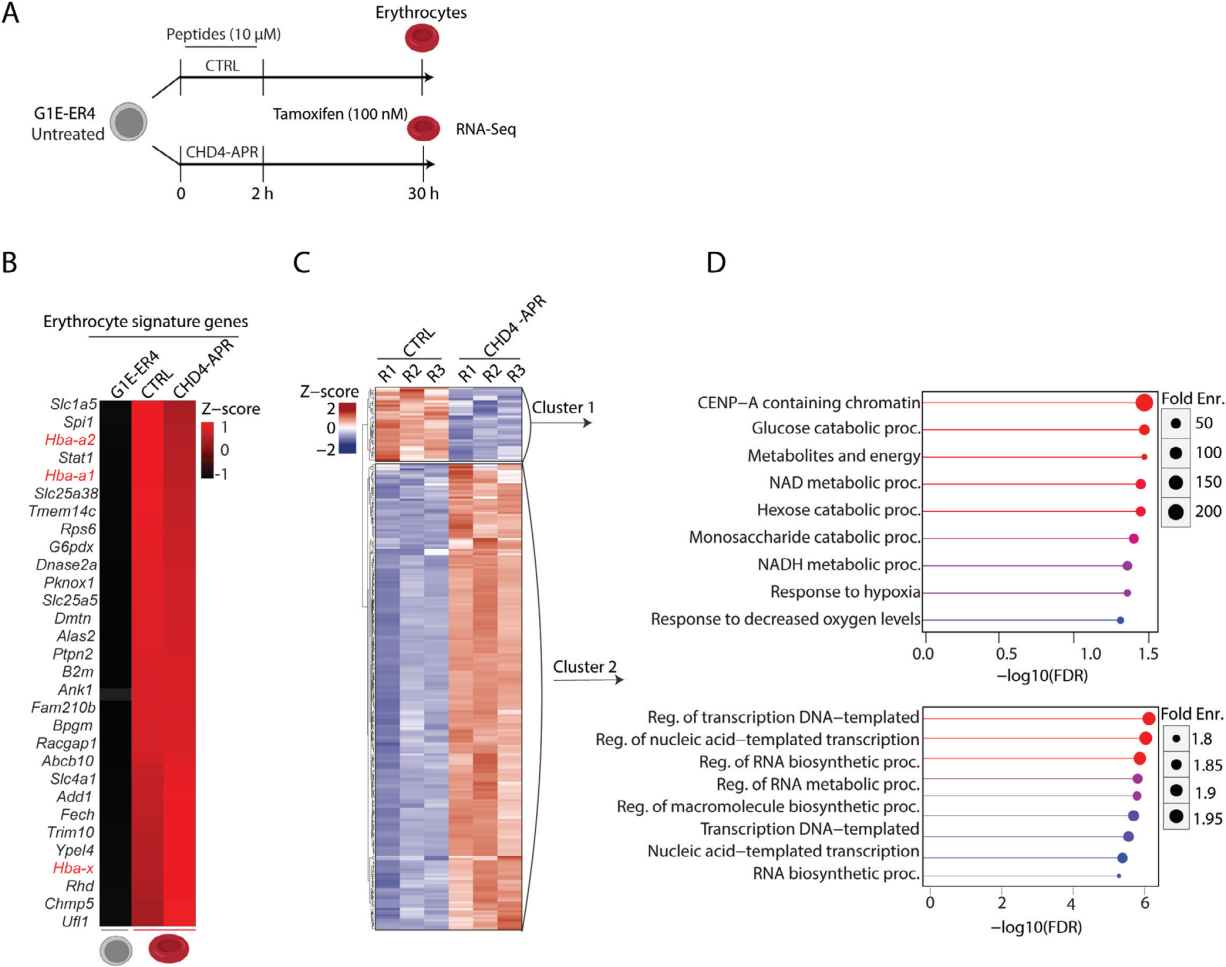
CHD4 dissociation from the NuRD and ChAHP complexes can lead to gene dysregulation. (A) Schematic illustrating the experimental procedure and sample collection timepoints. G1E‐ER4 cells were treated with either CHD4‐APR or CTRL peptides for 2 h and then tamoxifen was added, and samples were collected after 28 h (total 30 h). Erythrocytes were subjected to RNA‐Seq analysis. (B) Heatmap representing the upregulation of the erythrocytic signature genes in the presence of tamoxifen (top 30 genes are represented) and globin genes are highlighted in red. RNA‐Seq data of untreated G1E‐ER4 cells was obtained from ENCODE (GSE101195). The columns represent the mean of combined normalized expression from three replicates. (C) Z‐score heatmap representing the pattern of differentially expressed genes in CHD4‐APR versus CTRL peptides. (D) Gene ontology analysis indicating biological processes significantly associated with the differentially expressed genes.

Our results showed no significant differences in cell viability or apoptosis (Figure S3A,B). To investigate how the dissociation of CHD4 from the NuRD and ChAHP complexes affects the gene transcription program during erythropoiesis, we treated G1E‐ER4 cells with either CHD4‐APR or CTRL peptides (10 μM), followed by the induction of GATA‐1 expression and performed RNA‐Seq analysis (Figure 6A). Successful differentiation of G1E‐ER4 cells into erythrocytes following the induction of GATA‐1 was confirmed by increased expression of erythrocytic signature genes (e.g., *Hba‐a1*, *Hba‐a2*, and *Hba‐x*) compared to untreated G1E‐ER4 cells (Figure 6B). Differential gene expression analysis of CHD‐APR‐treated cells over CTRL peptide‐treated cells revealed significant dysregulation of 248 genes (Figure 6C). K‐means clustering of differentially expressed genes identified two distinct clusters (Figure 6C, Table S3). GO analysis of cluster 1 featuring downregulated genes upon CHD4‐APR treatment revealed enrichment of terms such as glucose catabolic processes and metabolites and energy‐producing processes. These processes ensure erythrocyte viability and function by providing energy, supporting lipid metabolism, and maintaining erythrocyte integrity. GO analysis of cluster 2 featuring upregulated genes revealed marked enrichment of terms including transcriptional regulation and RNA metabolism (Figure 6C). Taken together, our data support a critical interaction hub in CHD4 that facilitates and orchestrates a complex gene expression program leading to normal red blood cell production and metabolism.

## 4 Discussion

As CHD proteins are large (ranging in size from 197 to 336 kDa) and contain DNA and histone binding domains (such as ATPase and chromo domains), mapping protein interactions is labor intensive. Past attempts have often resulted in poor data quality. To overcome this issue, we focused on the N‐ and C‐termini of CHD family members and excluded the ATPase and chromo domains to minimize identification of indirect DNA‐ and histone‐mediated protein interactions. The AP‐MS approach typically requires the overexpression of bait proteins, but this may lead to their misexpression and/or mislocalization and thereby increasing the chance of false positives. To address these challenges, we first conducted nuclear fractionation to minimize interaction artifacts arising from protein mislocalization. Second, we standardized the expression levels of CHD proteins within a similar range by controlling DNA concentration and the number of transfected cells, as confirmed by iBAQ analysis. Additionally, indirect protein interactions bridged by DNA or RNA were minimized by sonicating samples to shear genomic DNA and treating the lysates with nucleases. Detecting known physical interactions, such as those between CHD subfamily I with RTF1 [38], and CHD3/CHD4/CHD5 interaction with the NuRD and ChAHP complexes verifies our AP‐MS workflow and reinforces the potential of our approach to identify novel binding partners.

We utilized Alphafold Multimer in conjunction with our AP‐MS data to characterize novel protein complexes. For several of these complexes, we observed high‐confidence PAE scores indicating a strong likelihood of direct interactions. However, the low confidence pLDDT scores assigned to intrinsically disordered portions of CHD suggest that Alphafold is limited in its ability to confidently predict the structural details of these interactions, which agrees with previous reports [42]. We also note that the N‐ and C‐termini of CHD proteins may adopt a tertiary structure under physiological conditions, particularly when CHD proteins engage with nucleosomes. Additionally, it is noteworthy that each protein chain within these complexes might also form homodimers or other higher‐order assemblies that are difficult to predict with Alphafold.

The novel CHD‐specific PPIs elucidated in this study provide a foundation for a more in‐depth exploration of the role of CHD proteins in chromatin biology, encompassing aspects such as gene expression, DNA replication, and repair mechanisms. The interaction of ANP32A, a chaperone crucial for H2A.Z deposition, with CHD6 suggests a role of this complex in the loading of H2A.Z in the regulatory regions of target genes [43]. The interaction of RSBN1 with multiple CHD proteins suggests functional synergy between CHDs, the histone mark H4K20me2, and DNA replication. H4K20me2 is involved in the recruitment of the origin recognition complex for the initiation of DNA replication [44]. The exclusive interaction of uncharacterized KIAA1671 protein with subfamily II CHDs may suggest a distinct biological role for this complex.

Our study provides clear evidence for the role of a unique C‐terminal CHD4 APR in coordinating the interaction of key functional proteins such as GATAD2B during erythropoiesis. Furthermore, the use of peptidomimetics as a tool to block or interfere with protein interactions mediated via APR‐containing interfaces emphasizes the utility of this approach. However, several limitations should be considered. First, in our RNA‐Seq experiment we used a low concentration of APR, which may have limited the inhibition of CHD4 engagement with the NuRD and ChAHP complexes, thereby limiting the degree of gene dysregulation. Timed collection of cells following tamoxifen induction could identify dynamic or temporal changes in gene expression during erythropoiesis. Despite these limitations, our study establishes intriguing paths for further investigations into the molecular mechanisms underlying CHD4 function.

The structures of CHD family proteins remain relatively poorly defined due to their large size and intrinsic flexibility arising from disordered sequences. Advances in cryogenic electron microscopy (cryo‐EM) and reconstituting nucleosomes and fragments of CHD proteins are gradually overcoming these challenges. Indeed, recent cryo‐EM studies have provided high‐resolution structures for nucleosome‐CHD1 and ‐CHD4 complexes [5, 45]. These structural studies are fundamental in improving our understanding of mechanisms of action of the CHD family of proteins. Our PPI data could be utilized to generate CHD‐N‐ and CHD‐C‐specific protein complexes, which can be produced recombinantly for cryo‐EM studies. This approach would enable detailed structural analysis of these complexes, providing insights into their functional mechanisms and relationships.

In conclusion, our study has identified the interaction networks of the CHD family of chromatin remodelers using proteomics and biochemical approaches. Importantly, our identification of a functional interaction hub within an IDR enhances our understanding of the complex regulatory mechanisms that govern the interplay between chromatin remodelers, chromatin dynamics, and gene expression.

## Author Contributions

M.S.T. and C.G.B. conceived the study, designed MS experiments, analyzed data, and wrote the manuscript. C.P. performed RNA‐Seq data analysis. C.G., Y.F., C.M., and R.N. assisted with cloning constructs, prepared samples for MS experiments, and performed western blots and MTT assays. A.C.H.W. conducted AlphaFold data analysis, and B.P.D. performed the flow cytometry analysis. J.E.J.R. reviewed the manuscript and provided intellectual input and scientific discussion.

## Conflicts of Interest

The authors declare no conflicts of interest.

## Supporting information


**Figure S1:** Bar graph showing iBAQ intensities for all FLAG‐CHD protein fragments used as a bait in AP‐MS experiments.

**Figure S2:** AlphaFold Multimer analysis of potential complexes. (A) Heatmaps represent the pattern of interaction and potential interaction interfaces highlighted in dotted rectangles. (B) 3D structures represent the confidence of the predicted structures.

**Figure S3:** Cell proliferation analysis. (A) Graph representing the MTT assay performed in three technical replicates (*n* = 3). Cell viability of G1E‐ER4 cells treated with a range of APR peptide concentrations was measured after 48 h. Data are presented as relative to CTRL peptide‐treated G1E‐ER4 cells. (B) Plot of G1E‐ER4 cells treated with 10 μM APR peptides for 2 h, treated with tamoxifen for 28 h, then stained with annexin V and DAPI and subjected to flow cytometry analysis.

Data S1.

Data S2.

Data S3.

## Data Availability

All raw MS data have been deposited to the ProteomeXchange Consortium via the PRIDE partner repository with the dataset identifier PXD055009. Raw RNA‐Seq data have been deposited in the NCBI gene expression omnibus (GEO) under accession number GSE275475.

## References

[1] J. Bednar , R. A. Horowitz , S. A. Grigoryev , et al., “Nucleosomes, Linker DNA, and Linker Histone Form a Unique Structural Motif That Directs the Higher‐Order Folding and Compaction of Chromatin,” Proceedings of the National Academy of Sciences of the United States of America 95 (1998): 14173–14178. 9826673 10.1073/pnas.95.24.14173 PMC24346

[2] W. K. M. Lai and B. F. Pugh , “Understanding Nucleosome Dynamics and Their Links to Gene Expression and DNA Replication,” Nature Reviews. Molecular Cell Biology 18 (2017): 548–562. 28537572 10.1038/nrm.2017.47 PMC5831138

[3] A. Saha , J. Wittmeyer , and B. R. Cairns , “Chromatin Remodelling: The Industrial Revolution of DNA Around Histones,” Nature Reviews. Molecular Cell Biology 7 (2006): 437–447. 16723979 10.1038/nrm1945

[4] C. R. Clapier , J. Iwasa , B. R. Cairns , and C. L. Peterson , “Mechanisms of Action and Regulation of ATP‐Dependent Chromatin‐Remodelling Complexes,” Nature Reviews. Molecular Cell Biology 18 (2017): 407–422. 28512350 10.1038/nrm.2017.26 PMC8127953

[5] L. Farnung , M. Ochmann , and P. Cramer , “Nucleosome‐CHD4 Chromatin Remodeler Structure Maps Human Disease Mutations,” eLife 9 (2020): e56178. 32543371 10.7554/eLife.56178 PMC7338049

[6] D. C. Hargreaves and G. R. Crabtree , “ATP‐Dependent Chromatin Remodeling: Genetics, Genomics and Mechanisms,” Cell Research 21 (2011): 396–420. 21358755 10.1038/cr.2011.32 PMC3110148

[7] Y. Zhong , B. P. Paudel , D. P. Ryan , et al., “CHD4 Slides Nucleosomes by Decoupling Entry‐ and Exit‐Side DNA Translocation,” Nature Communications 11 (2020): 1519. 10.1038/s41467-020-15183-2 PMC7090039 32251276

[8] J. K. K. Low , A. P. G. Silva , M. Sharifi Tabar , et al., “The Nucleosome Remodeling and Deacetylase Complex has an Asymmetric, Dynamic, and Modular Architecture,” Cell Reports 33 (2020): 108450. 33264611 10.1016/j.celrep.2020.108450 PMC8908386

[9] V. Ostapcuk , F. Mohn , S. H. Carl , et al., “Activity‐Dependent Neuroprotective Protein Recruits HP1 and CHD4 to Control Lineage‐Specifying Genes,” Nature 557 (2018): 739–743. 29795351 10.1038/s41586-018-0153-8

[10] M. Sharifi Tabar , C. Giardina , Y. Feng , et al., “Unique Protein Interaction Networks Define the Chromatin Remodelling Module of the NuRD Complex,” FEBS Journal 289 (2022): 199–214. 34231305 10.1111/febs.16112 PMC9545347

[11] M. Sharifi Tabar , J. P. Mackay , and J. K. K. Low , “The Stoichiometry and Interactome of the Nucleosome Remodeling and Deacetylase (NuRD) Complex Are Conserved Across Multiple Cell Lines,” FEBS Journal 286 (2019): 2043–2061. 30828972 10.1111/febs.14800

[12] C. G. Marfella and A. N. Imbalzano , “The CHD Family of Chromatin Remodelers,” Mutation Research 618 (2007): 30–40. 17350655 10.1016/j.mrfmmm.2006.07.012 PMC1899158

[13] J. T. Trujillo , J. Long , E. Aboelnour , J. Ogas , and J. H. Wisecaver , “CHD Chromatin Remodeling Protein Diversification Yields Novel Clades and Domains Absent in Classic Model Organisms,” Genome Biology and Evolution 14 (2022): evac066. 35524943 10.1093/gbe/evac066 PMC9113485

[14] H. Durr , A. Flaus , T. Owen‐Hughes , and K. P. Hopfner , “Snf2 Family ATPases and DExx Box Helicases: Differences and Unifying Concepts From High‐Resolution Crystal Structures,” Nucleic Acids Research 34 (2006): 4160–4167. 16935875 10.1093/nar/gkl540 PMC1616948

[15] E. J. Enemark and L. Joshua‐Tor , “On Helicases and Other Motor Proteins,” Current Opinion in Structural Biology 18 (2008): 243–257. 18329872 10.1016/j.sbi.2008.01.007 PMC2396192

[16] A. Patil , A. R. Strom , J. A. Paulo , et al., “A Disordered Region Controls cBAF Activity via Condensation and Partner Recruitment,” Cell 186, no. e26 (2023): 4936–4955. 37788668 10.1016/j.cell.2023.08.032 PMC10792396

[17] M. Sharifi Tabar , C. Parsania , H. Chen , X. D. Su , C. G. Bailey , and J. E. J. Rasko , “Illuminating the Dark Protein‐Protein Interactome,” Cell Reports Methods 2 (2022): 100275. 36046620 10.1016/j.crmeth.2022.100275 PMC9421580

[18] M. Rylski , J. J. Welch , Y. Y. Chen , et al., “GATA‐1‐Mediated Proliferation Arrest During Erythroid Maturation,” Molecular and Cellular Biology 23 (2003): 5031–5042. 12832487 10.1128/MCB.23.14.5031-5042.2003 PMC162202

[19] A. D. Shah , R. J. A. Goode , C. Huang , D. R. Powell , and R. B. Schittenhelm , “LFQ‐Analyst: An Easy‐To‐Use Interactive Web Platform to Analyze and Visualize Label‐Free Proteomics Data Preprocessed With MaxQuant,” Journal of Proteome Research 19 (2020): 204–211. 31657565 10.1021/acs.jproteome.9b00496

[20] R. Evans , M. O'Neill , A. Pritzel , et al., “Protein Complex Prediction With AlphaFold‐Multimer,” 2022 2021.10.04.463034.

[21] J. Jumper , R. Evans , A. Pritzel , et al., “Highly Accurate Protein Structure Prediction With AlphaFold,” Nature 596 (2021): 583–589. 34265844 10.1038/s41586-021-03819-2 PMC8371605

[22] A. M. Fernandez‐Escamilla , F. Rousseau , J. Schymkowitz , and L. Serrano , “Prediction of Sequence‐Dependent and Mutational Effects on the Aggregation of Peptides and Proteins,” Nature Biotechnology 22 (2004): 1302–1306. 10.1038/nbt1012 15361882

[23] A. Dobin , C. A. Davis , F. Schlesinger , et al., “STAR: Ultrafast Universal RNA‐Seq Aligner,” Bioinformatics 29 (2013): 15–21. 23104886 10.1093/bioinformatics/bts635 PMC3530905

[24] A. Kechin , U. Boyarskikh , A. Kel , and M. Filipenko , “cutPrimers: A New Tool for Accurate Cutting of Primers From Reads of Targeted Next Generation Sequencing,” Journal of Computational Biology 24 (2017): 1138–1143. 28715235 10.1089/cmb.2017.0096

[25] Y. Liao , G. K. Smyth , and W. Shi , “The R Package Rsubread is Easier, Faster, Cheaper and Better for Alignment and Quantification of RNA Sequencing Reads,” Nucleic Acids Research 47 (2019): e47. 30783653 10.1093/nar/gkz114 PMC6486549

[26] M. I. Love , W. Huber , and S. Anders , “Moderated Estimation of Fold Change and Dispersion for RNA‐Seq Data With DESeq2,” Genome Biology 15 (2014): 550. 25516281 10.1186/s13059-014-0550-8 PMC4302049

[27] C. Parsania , R. Chen , P. Sethiya , Z. Miao , L. Dong , and K. H. Wong , “FungiExpresZ: An Intuitive Package for Fungal Gene Expression Data Analysis, Visualization and Discovery,” Briefings in Bioinformatics 24 (2023): bbad051. 36806894 10.1093/bib/bbad051 PMC10025439

[28] S. X. Ge , D. Jung , and R. Yao , “ShinyGO: A Graphical Gene‐Set Enrichment Tool for Animals and Plants,” Bioinformatics 36 (2020): 2628–2629. 31882993 10.1093/bioinformatics/btz931 PMC7178415

[29] A. P. Silva , D. P. Ryan , Y. Galanty , et al., “The N‐Terminal Region of Chromodomain Helicase DNA‐Binding Protein 4 (CHD4) is Essential for Activity and Contains a High Mobility Group (HMG) Box‐Like‐Domain That Can Bind Poly(ADP‐Ribose),” Journal of Biological Chemistry 291, no. 2 (2016): 924–938. 26565020 10.1074/jbc.M115.683227 PMC4705410

[30] J. J. Lin , L. W. Lehmann , G. Bonora , et al., “Mediator Coordinates PIC Assembly With Recruitment of CHD1,” Genes & Development 25 (2011): 2198–2209. 21979373 10.1101/gad.17554711 PMC3205589

[31] Y. J. Joo , S. B. Ficarro , L. M. Soares , Y. Chun , J. A. Marto , and S. Buratowski , “Downstream Promoter Interactions of TFIID TAFs Facilitate Transcription Reinitiation,” Genes & Development 31 (2017): 2162–2174. 29203645 10.1101/gad.306324.117 PMC5749164

[32] S. C. Dillon , X. Zhang , R. C. Trievel , and X. Cheng , “The SET‐Domain Protein Superfamily: Protein Lysine Methyltransferases,” Genome Biology 6 (2005): 227. 16086857 10.1186/gb-2005-6-8-227 PMC1273623

[33] K. Brejc , Q. Bian , S. Uzawa , et al., “Dynamic Control of X Chromosome Conformation and Repression by a Histone H4K20 Demethylase,” Cell 171 (2017): 85–102.e23. 28867287 10.1016/j.cell.2017.07.041 PMC5678999

[34] C. Borgo , C. D'Amore , S. Sarno , M. Salvi , and M. Ruzzene , “Protein Kinase CK2: A Potential Therapeutic Target for Diverse Human Diseases,” Signal Transduction and Targeted Therapy 6, no. 1 (2021): 183. 33994545 10.1038/s41392-021-00567-7 PMC8126563

[35] S. Brahma and S. Henikoff , “The BAF Chromatin Remodeler Synergizes With RNA Polymerase II and Transcription Factors to Evict Nucleosomes,” Nature Genetics 56 (2024): 100–111. 38049663 10.1038/s41588-023-01603-8 PMC10786724

[36] C. Jeronimo , A. Angel , V. Q. Nguyen , et al., “FACT is Recruited to the +1 Nucleosome of Transcribed Genes and Spreads in a CHD1‐Dependent Manner,” Molecular Cell 81 (2021): 3542–3559.e11. 34380014 10.1016/j.molcel.2021.07.010 PMC9149603

[37] L. Farnung , M. Ochmann , M. Engeholm , and P. Cramer , “Structural Basis of Nucleosome Transcription Mediated by CHD1 and FACT,” Nature Structural & Molecular Biology 28 (2021): 382–387. 10.1038/s41594-021-00578-6 PMC8046669 33846633

[38] R. Simic , D. L. Lindstrom , H. G. Tran , et al., “Chromatin Remodeling Protein CHD1 Interacts With Transcription Elongation Factors and Localizes to Transcribed Genes,” EMBO Journal 22 (2003): 1846–1856. 12682017 10.1093/emboj/cdg179 PMC154471

[39] M. Torrado , J. K. K. Low , A. P. G. Silva , et al., “Refinement of the Subunit Interaction Network Within the Nucleosome Remodelling and Deacetylase (NuRD) Complex,” FEBS Journal 284 (2017): 4216–4232. 29063705 10.1111/febs.14301 PMC5734987

[40] J. Abramson , J. Adler , J. Dunger , et al., “Accurate Structure Prediction of Biomolecular Interactions With AlphaFold 3,” Nature 630 (2024): 493–500. 38718835 10.1038/s41586-024-07487-w PMC11168924

[41] K. M. Ruff and R. V. Pappu , “AlphaFold and Implications for Intrinsically Disordered Proteins,” Journal of Molecular Biology 433 (2021): 167208. 34418423 10.1016/j.jmb.2021.167208

[42] H. Bret , J. Gao , D. J. Zea , J. Andreani , and R. Guerois , “From Interaction Networks to Interfaces, Scanning Intrinsically Disordered Regions Using AlphaFold2,” Nature Communications 15 (2024): 597. 10.1038/s41467-023-44288-7 PMC10796318 38238291

[43] Z. Mao , L. Pan , W. Wang , et al., “Anp32e, a Higher Eukaryotic Histone Chaperone Directs Preferential Recognition for H2A.Z,” Cell Research 24 (2014): 389–399. 24613878 10.1038/cr.2014.30 PMC3975505

[44] L. Huang , Y. Wang , H. Long , et al., “Structural Insight Into H4K20 Methylation on H2A.Z‐Nucleosome by SUV420H1,” Molecular Cell 83 (2023): 2884–2895.e7. 37536340 10.1016/j.molcel.2023.07.001

[45] L. Farnung , S. M. Vos , C. Wigge , and P. Cramer , “Nucleosome‐CHD1 Structure and Implications for Chromatin Remodelling,” Nature 550 (2017): 539–542. 29019976 10.1038/nature24046 PMC5697743

