# Supplementary figures and images for: Intrinsically Disordered Regions Define Unique Protein Interaction Networks in CHD Family Remodelers
Source: FASEB J. 2025 May 15;39(10):e70632. doi: 10.1096/fj.202402808RR (PMC12080455; doi:10.1096/fj.202402808RR)

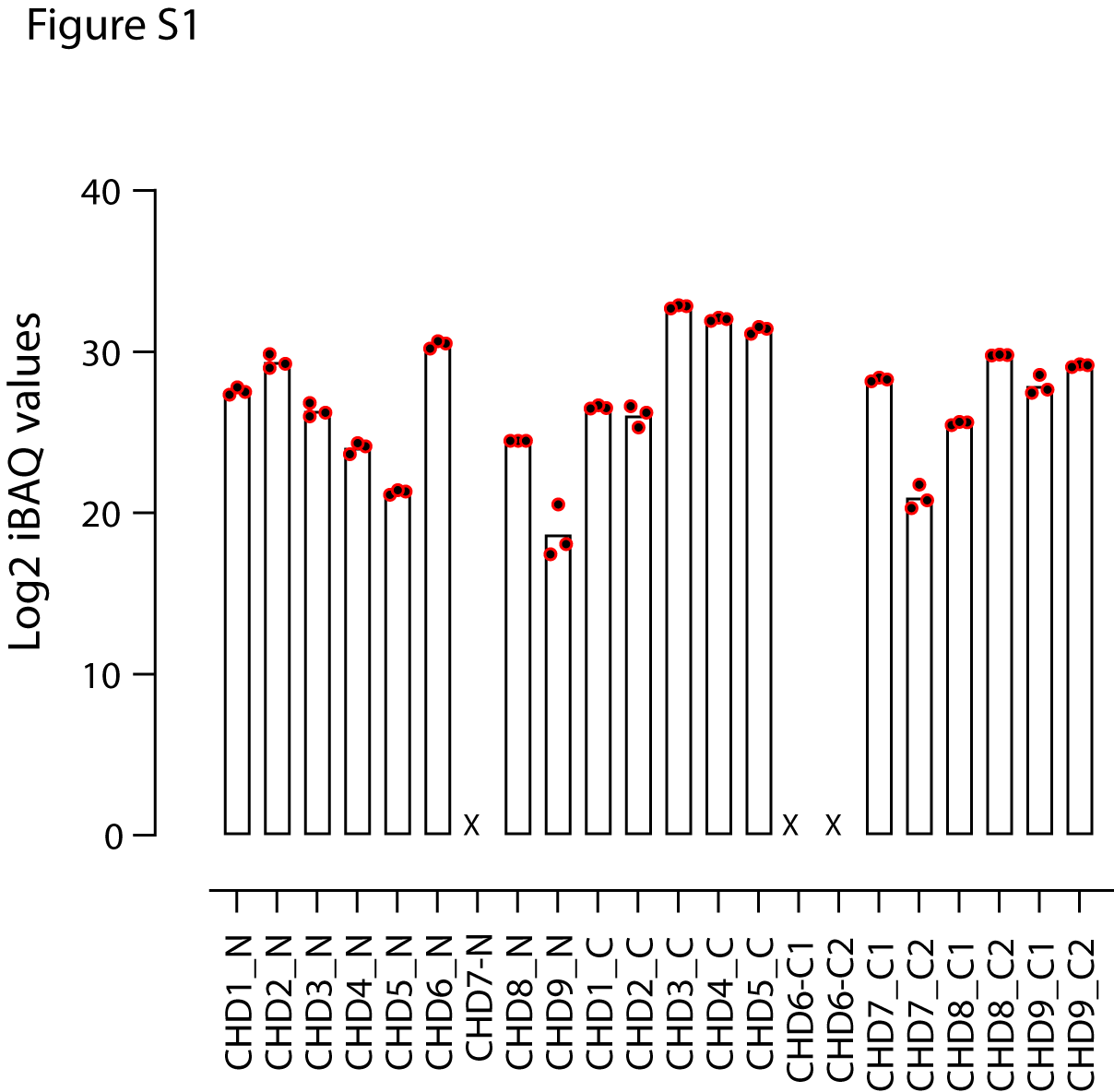

Supplement: Supplementary file 1 — Figure S1: Bar graph showing iBAQ intensities for all FLAG‐CHD protein fragments used as a bait in AP‐MS experiments. [file FSB2-39-e70632-s005.tif]

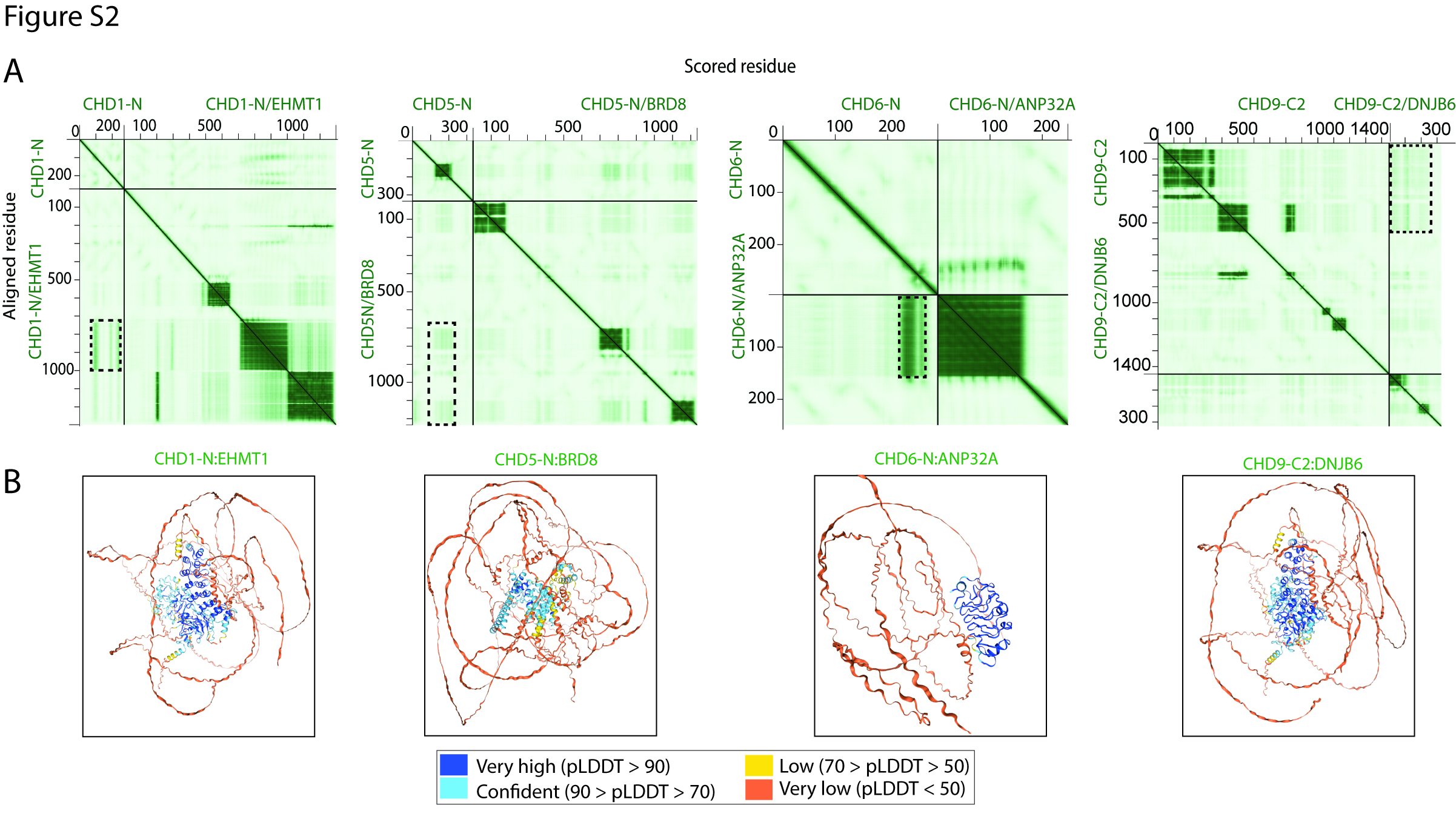

Supplement: Supplementary file 2 — Figure S2: AlphaFold Multimer analysis of potential complexes. (A) Heatmaps represent the pattern of interaction and potential interaction interfaces highlighted in dotted rectangles. (B) 3D structures represent the confidence of the predicted structures. [file FSB2-39-e70632-s004.tif]

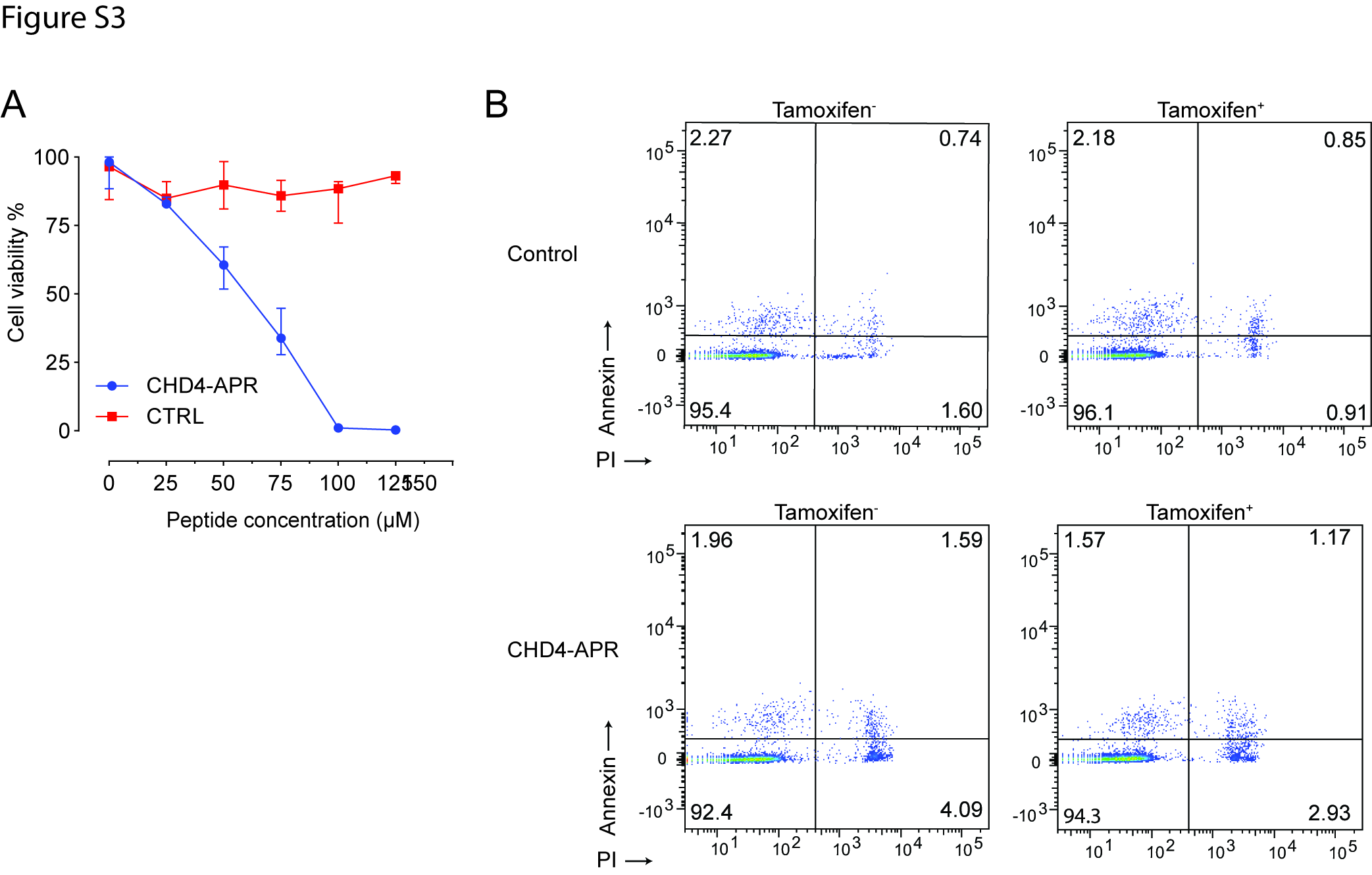

Supplement: Supplementary file 3 — Figure S3: Cell proliferation analysis. (A) Graph representing the MTT assay performed in three technical replicates (n = 3). Cell viability of G1E‐ER4 cells treated with a range of APR peptide concentrations was measured after 48 h. Data are presented as relative to CTRL peptide‐treated G1E‐ER4 cells. (B) Plot of G1E‐ER4 cells treated with 10 μM APR peptides for 2 h, treated with tamoxifen for 28 h, then stained with annexin V and DAPI and subjected to flow cytometry analysis. [file FSB2-39-e70632-s003.tif]
